# Spatio-Temporal Variation and Futuristic Emission Scenario of Ambient Nitrogen Dioxide over an Urban Area of Eastern India Using GIS and Coupled AERMOD–WRF Model

**DOI:** 10.1371/journal.pone.0170928

**Published:** 2017-01-31

**Authors:** Sharadia Dey, Srimanta Gupta, Precious Sibanda, Arun Chakraborty

**Affiliations:** 1 School of Mathematics, Statistics and Computer Science, University of KwaZulu-Natal, Private Bag X01 Scottsville, Pietermaritzburg, South Africa; 2 Department of Environmental Science, The University of Burdwan, Golapbag, Burdwan, West Bengal, India; 3 Center for Oceans, Rivers, Atmosphere and Land Sciences (CORAL), Indian Institute of Technology, Kharagpur, West Bengal, India; Beihang University, CHINA

## Abstract

The present study focuses on the spatio-temporal variation of nitrogen dioxide (NO_2_) during June 2013 to May 2015 and its futuristic emission scenario over an urban area (Durgapur) of eastern India. The concentration of ambient NO_2_ shows seasonal as well as site specific characteristics. The site with high vehicular density (Muchipara) shows highest NO_2_ concentration followed by industrial site (DVC- DTPS Colony) and the residential site (B Zone), respectively. The seasonal variation of ambient NO_2_ over the study area is portrayed by means of Geographical Information System based Digital Elevation Model. Out of the total urban area under consideration (114.982 km^2^), the concentration of NO_2_ exceeded the National Ambient Air Quality Standard (NAAQS) permissible limit over an area of 5.000 km^2^, 0.786 km^2^ and 0.653 km^2^ in post monsoon, winter and pre monsoon, respectively. Wind rose diagrams, correlation and regression analyses show that meteorology plays a crucial role in dilution and dispersion of NO_2_ near the earth’s surface. Principal component analysis identifies vehicular source as the major source of NO_2_ in all the seasons over the urban region. Coupled AMS/EPA Regulatory Model (AERMOD)–Weather Research and Forecasting (WRF) model is used for predicting the concentration of NO_2_. Comparison of the observed and simulated data shows that the model overestimates the concentration of NO_2_ in all the seasons (except winter). The results show that coupled AERMOD–WRF model can overcome the unavailability of hourly surface as well as upper air meteorological data required for predicting the pollutant concentration, but improvement of emission inventory along with better understanding of the sinks and sources of ambient NO_2_ is essential for capturing the more realistic scenario.

## Introduction

The surface emission sources and patterns of major air pollutants have been substantially changing over the tropical region. Rapid urbanization has led to an increasing number of large population agglomerations. Gradual degradation of air quality is one of the negative outcomes of modernization on human beings and environment. Escalating air pollution in urban areas is a matter of concern worldwide. The increasing levels of gaseous air pollutants pose a serious risk to human health and environment due to their detrimental effects. Nitrogen dioxide (NO_2_) is one of the criteria pollutants identified by Clean Air Act of 1970. It is an important trace gas which has a potential direct role in global climate change and plays a central role in tropospheric chemistry. It acts as a precursor for a number of harmful secondary air pollutants such as tropospheric ozone (O_3_) and plays a crucial role in the formation of acid rain. In the troposphere, nitric oxide (NO) is mainly emitted which in turn is rapidly converted to NO_2_. During daytime, a steady state is established NO and NO_2_ leading to the formation of tropospheric O_3_. The residence time of NO_2_ in the atmosphere is found to be approximately 0.5–2 days. Current scientific evidence links short-term NO_2_ exposures (ranging from 30 minutes to 24 hours) with adverse respiratory effects including airway inflammation in healthy people, increased respiratory symptoms in peoples suffering from asthma and increased epilepsy attack [1]. Oxides of nitrogen i.e. NO_x_ (including NO_2_) and volatile organic compounds react in the presence of heat and sunlight to form O_3_ which in turn causes reduction in lung function, aggravation of pre-existing respiratory disease (such as asthma), increased daily hospital admissions and emergency department visits for respiratory causes and excess mortality.

The increasing levels of NO_2_ and NO_x_ especially in the urban areas have gained attention worldwide. Along with other gaseous pollutants, NO_2_ and NO_x_ were monitored and analyzed in in Pakistan [2], Al-Ain city, UAE [3], Metropolitan area of Monterrey, Mexico [4] etc. Investigation of the concentration of NO_2_ and NO_x_ were carried out in different spatial and temporal scale in different corners of India like Lucknow, Haryana, Kolkata, Delhi, Burdwan and Gopalpur [5–10]. Zhao et al. [11] explored the association of higher concentration of ambient NO_2_ with high ozone days (HODs) over Shanghai, China. The interaction of multiple sources and various processes in different spatial and temporal scales make the urban air quality modeling more complicated. Borge et al. [12] performed a comprehensive source apportionment study in the Madrid metropolitan area by using a multi-scale, multi-pollutant air quality modeling system (WRF- SMOKE-CMAQ). He et al. [13–14] predicted particulate matters at urban area by using coupled artificial neural network—chaotic particle swarm optimization algorithm as well as by hybrid model combining multi layer perceptron model and principal component analysis. Several researches have been performed on driver’s bound rationality, fuel consumption and emissions [15–16]. Dispersion of a pollutant is a complex function of meteorological factors, planetary boundary layer characteristics and interactions with other species present in the ambient air. Therefore, quality data of these parameters are required as inputs to the dispersion models used for modeling the urban air quality status. Rao et al. [17] and Sharma et al. [18] evaluated the performance and predictive capacity of some commonly used dispersion models and concluded that these models are consistence with the dynamic nature of the atmosphere and are suitable for exploring the dispersion of pollutants. Such dispersion models are increasingly used for forecasting urban air quality status [19–20] and the necessary meteorological inputs are generated using suitable prognostic models like e.g. MM5 [21], Eta [19], WRF [20] etc. Weather Research and Forecasting (WRF) can successfully generate the meteorological inputs required for AERMOD [20].

The study area (Durgapur) has witnessed rapid industrialization and urbanization in the last few decades and it is known to be one of the most polluted urban areas of the country. Deteriorating air quality scenario of this urban area has posed a serious risk to human health and environment due to their detrimental effects. To the best of authors’ knowledge, spatio–temporal variation of air pollutants along with its future projection has not yet taken place over this region. Such a study is essential for formulation and effective implementation of air pollution abatement measures. The focus of this study is on the spatial and temporal variation of the NO_2_ over this tropical urban area (Durgapur) and to obtain futuristic emission scenario over this region. The spatial and temporal variation of NO_2_ is obtained by using Geographical Information System (GIS) based Digital Elevation Model (GeomaticaV.10.1). A Gaussian air pollutant dispersion model AMS/EPA Regulatory Model or AERMOD [22] is used for understanding the dispersion of NO_2_ over the chosen area. AERMOD needs hourly surface and upper air meteorological observations for simulating the pollutant dispersion which is not available over this urban area. A high resolution prognostic model, Weather Research and Forecasting (WRF), is used for generating the required meteorological data. Coupled WRF–AERMOD simulates the present as well as future emission scenario of NO_2_ over the urban area. Finally, a comparative study of the simulated and observed values of NO_2_ at different sites over the urban is performed.

## Data and Method

### Description of the study area

**Fig 1:**
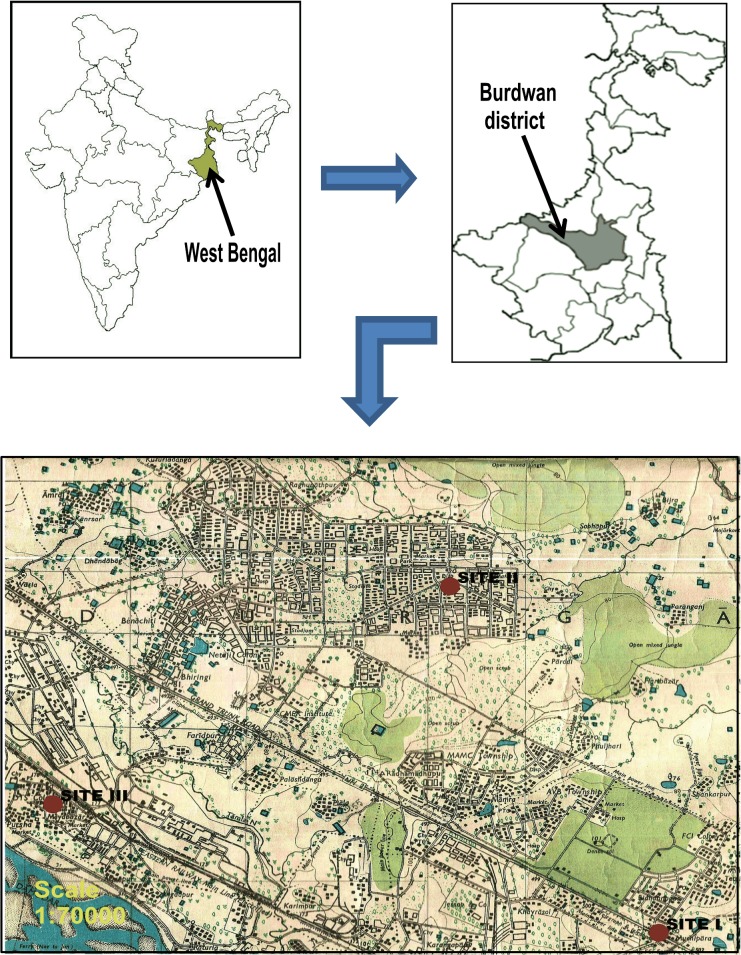
Description of the study area.

Durgapur (chosen urban area) is situated in the Burdwan district of West Bengal, India. It is located on the bank of River Damodar. This area is covered with Red and Yellow Ultisols soil and the topography of this area is undulating, with an average elevation of 65 m MSL. This area experiences a transitional climate between the tropical wet and dry climate and the more humid subtropical climate. Three different sites are selected in the urban area for sampling (denoted by red dots in Fig 1).The sites are located in private ownership areas, so no specific permissions are required for the sampling activities in the chosen sites. Moreover, the field studies do not involve any endangered or protected species. The details of these sites are as follows:

Site I (Muchipara) is situated at 23° 30 ′13.79 ″ N and 87° 21′16.82 ″ E. The sampling site is adjacent to National High Way (NH-2) or the Grand Trunk Road. This site represents an area with high vehicular density.
Site II (B Zone) is located at 23°33′ 54.21ʺ N and 87°19′16.22ʺ E. This site is situated in a residential area which is approximately 4.1 km from national highway and 7.5 km industrial area.
Site III (DVC-DTPS Colony) is situated in an industrial area at 23° 31′ 35.11ʺ N and 87° 15′ 27.94ʺ E. River Damodar flows to its south. The Durgapur Steel Plant (DSP) and the Durgapur Thermal Power Station (DTPS) of the Damodar Valley Corporation (DVC) are close to this site.

### Data collection

High Volume Sampler (Envirotech APM 460BL) is used for 24 hour sampling and the concentration of ambient NO_2_ is determined by Modified Jacobs and Hochheiser Method [23]. Relative humidity and temperature are measured by a portable hygrometer (Model-HTC-1), wind speed is measured by a digital anemometer (Model-Lutron-AM-4201) and wind direction is recorded by a wind vane. The meteorological parameters are recorded at a regular interval of 1 hour.

The data of different criteria pollutants are collected from the Durgapur Station of West Bengal Pollution Control Board (www.wbpcb.gov.in).

### Statistical analyses

#### Correlation analysis

Pearson correlation coefficients between NO_2_ and different meteorological parameters are calculated by using the formula

$$r=\frac{\sum_{i=1}^{n}\left(X_{i}-\bar{X})(Y_{i}-\bar{Y}\right)}{(n-1)S_{x}S_{y}}$$ (1)

Where, *X* and *Y* are two variables, with means $\bar{X}$ and $\bar{Y}$ respectively and with standard deviations *S*_*x*_ and *S*_*y*_ respectively.

#### Regression analysis

Multiple linear regression (MLR) attempts to model the relationship between two or more explanatory variables (independent variables) and a response variable (dependent variable) by fitting a linear equation to observed data. MLR technique has the capability of exploring the contribution of selected variables to chosen air pollutant concentration. The general equation of MLR is expressed as [24]

$$y=b_{0}+\sum_{k=1}^{p}b_{i}x_{i}+\xi$$ (2)

where,

b_i_ is the regression coefficient,

x_i_ is the independent variable, and

ξ is the stochastic error associated with the regressions.

#### Principal component analysis (PCA)

Among multivariate techniques, Principal components analysis (PCA) is designed to classify variables based on their correlations with each other. The goal of PCA is to consolidate a large number of observed variables into a smaller number of factors (components) that can be more readily interpreted as these underlying processes. It is often used as an exploratory tool to identify the major sources of air pollutant emissions [25–26].

In general, principal components (PCs) are expressed by the following equation

$$PC_{i}=A_{1i}V_{i}+A_{2i}V_{2}+\ldots \ldots .+A_{ni}V_{n}$$ (3)

where,

PC_i_ is principal component i and

A_ni_ is the loading (correlation coefficient) of the original variable V_n_

All the statistical analyses are performed by using XLSTAT 2010.

### Model description

#### GIS based digital elevation model

Digital Elevation Model (DEM) has been used as an effective tool for exploring the spatial and temporal variation of air pollutants in a GIS environment. DEM is generated on the basis of sampling points stored as point layer along with the NO_2_ by using VEDIMINT algorithm in the Geomatica V.10.1. The output of DEM is represented as a zonation map of the NO_2_ which gives an idea of the spatial distribution of the NO_2_ in four different seasons (i.e. monsoon, post monsoon, winter and pre monsoon) over the study area.

#### Numerical modeling

**Dispersion model (AERMOD):** It is a steady-state Gaussian plume model useful for the computation of pollutant dispersion applicable for multiple sources (point, area and volume) of emissions in rural and urban areas [22, 27–28]. This model accounts for the vertical in- homogeneity of the PBL in its dispersion calculation by averaging the parameters of actual PBL into effective parameters of an equivalent homogeneous planetary boundary layer (PBL). The PBL parameters such as friction velocity, Monin—Obukhov length, convective velocity scale, temperature scale, mixing height, surface heat flux are computed by AERMET (meteorological preprocessor of AERMOD) by using local surface characteristics in the form of surface roughness and Bowen ratio in combination with standard meteorological observations (wind speed, wind direction, temperature and cloud cover). These obtained parameters are then passed through an interface present in AERMOD for calculating vertical profiles of wind speed, lateral and vertical turbulent fluctuations and potential temperature gradient. AERMOD is found to be useful for simulation of short–range (less than 50 km) pollutant dispersion especially for urban areas.

Hourly surface as well as upper air meteorological observations are required along with the emission inventory to integrate AERMOD model. But unfortunately, such kind of meteorological data which is essential for the computation of the required boundary layer parameters that serve as input to AERMOD is not available over the study area. The surface parameters and PBL parameters which serve as input in AERMOD are obtained from the output of WRF model.

#### Gridded emission inventory

Preparation of emission inventory is an indispensible scientific tool for prevention of air pollution and air quality management. Quantitative understanding of the emission helps in better identification of the actual emission sources and estimation of the future emission scenario. The calculation of emissions from vehicles over the study area is performed on the basis of emission factors for different types of vehicles according to Automotive Research Association of India [29], number of vehicles of specific type, the distance travelled by a particular vehicle and their distribution based on the type of the fuel used. The estimation of vehicular emission is based on earlier works [30–32],

$$E_{i}=\sum (Veh_{j}\times D_{j})\times E_{i;j;km}$$ (4)

where E_i_ is the emission of compound (*i*), Veh_j_ is the number of vehicles per type (j), *D*_j_ is the distance travelled in a year per different vehicle type (j) and *E*_i;j;km_ is the emission factor of compound (*i*) of vehicle type (j) per driven kilometer.

The obtained gridded emission inventory serves as one of the inputs of AERMOD.

#### Mesoscale atmospheric model

The Advanced Research Weather Research and Forecasting (WRF—ARW) model using Eulerian Mass Dynamical core developed by National Center for Atmospheric Research (NCAR) is a flexible, state-of-the-art atmospheric simulation system which can be used for research work in different spatial scales ranging from meters to kilometers. A detailed description of the model physics, equations and dynamics is available in Skamarock et al. [33]. In this model, eight PBL schemes are available for parameterization of the sub-grid scale turbulent vertical fluxes of heat, moisture and momentum within the PBL. Out of eight available PBL schemes of WRF model, YSU i.e. Yonsei University [34] scheme is found to give good performance over urban area and is useful for air pollution dispersion studies [35]. So this PBL scheme is used for the future projection of air pollutant in the present study. This model is run using 1° X 1° resolution with 6 hourly National Centre for Environmental Prediction (NCEP) Final Analysis (FNL) data for the initial and boundary conditions.

The mesoscale atmospheric model (WRF) and dispersion model (AERMOD) are coupled for obtaining the futuristic emission scenario over the chosen urban area.

## Result and Discussion

### Seasonal variation of NO_2_ over the study area

**Fig 2:**
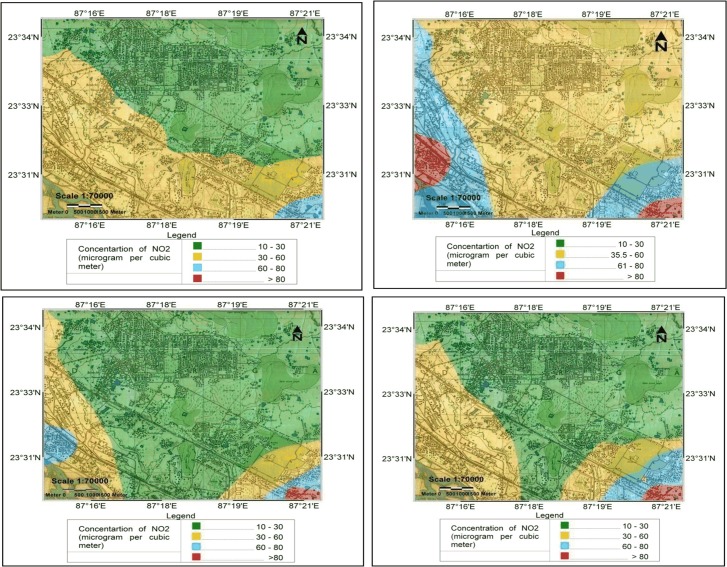
Spatial distribution of NO_2_ over the urban area during (a) monsoon season, (b) post monsoon, (c) winter and (d) pre monsoon seasons.

GIS based DEM model is used for obtaining the spatial distribution of NO_2_ over the urban area in four different seasons. During the monsoon season, the average concentrations of NO_2_ are found to be 63.795 μg/m^3^, 11.100 μg/m^3^ and 35.693 μg/m^3^ in Site I, Site II and Site III respectively. The concentration of NO_2_ lies between 10–30 μg/m^3^ over 59.434 km^2^, between 30–60 μg/m^3^ over 53.624 km^2^ and between 60–80 μg/m^3^ over 1.924 km^2^ area of the urban region as shown in Fig 2(A). Shallow PBL height, low temperature and pressure leads to accumulation of air pollutants near the earth’s surface. These combines effects meteorology and PBL result in the increase of pollutant loads near the ground in both post monsoon and winter seasons. The total area of the urban region under consideration is 114.982 km^2^. The DEM of spatial distribution of NO_2_ over the urban area for post monsoon season [Fig 2(B)] shows that the concentration of NO_2_ lies between 30–60 μg/m^3^ over 87.036 km^2^ and between 60–80 μg/m^3^ over 22.946 km^2^ area of the urban region under consideration. The concentration of NO_2_ is above the permissible limit of NAAQS (*i*.*e*. above 80 μg/m^3^) over an area 5.000 km^2^. In winter season, a large portion of the urban area (86.573 km^2^) is covered by the concentration range of 10–30 μg/m^3^. 23.38 km^2^ and 4.243 km^2^ are covered by the concentration range of 30–60 μg/m^3^ and 60–80 μg/m^3^ respectively **[**Fig 2(C)]. The NAAQS permissible limit for 24 h NO_2_ concentration is exceeded over an area of 0.786 km^2^. The average concentrations (ranges) of NO_2_ concentration are found to be 182.836 μg/m^3^ (84.490–315.651 μg/m^3^), 60.605 μg/m^3^ (35.173–98.604 μg/m^3^) and 135.123 μg/m^3^ (87.786–229.204 μg/m^3^) at Site I, Site II and Site III respectively. The concentration of NO_2_ is highest over Site I followed by Site III and Site II. The pattern of spatial distribution of NO_2_ gradually changes with the advent of pre monsoon season [Fig 2(D)]. The average concentrations (ranges) of NO_2_ are found to be 93.591 μg/m^3^ (67.955–145.575 μg/m^3^), 29.149 μg/m^3^ (10.092–51.899 μg/m^3^) and 61.915 μg/m^3^ (34.147–129.334 μg/m^3^) at Site I, Site II and Site III respectively. Increase in PBL height leads to gradual dispersion of pollutants which in turns dilutes the pollutant concentration near the earth’s surface. In this season, 74.815 km^2^ lies in the range of 10–30 μg/m^3^, 36.122 km^2^ lies in the 30–60 μg/m^3^ category and 3.392 km^2^ lies in 60–80 μg/m^3^ concentration range. The concentration of NO_2_ is above the NAAQS permissible limit over 0.653 km^2^ only during pre monsoon season.

### Comparison of concentration of NO_2_ with other urban areas

The NO_2_ level at a residential area of Kolkata, India was to be 32.500±14.200 μg/m^3^ whereas in the industrial area, the concentration of NO_2_ was 49.900±9.800 μg/m^3^ [7]. The average concentration of NO_2_ was found to be 10.70 ± 3.25 ppb (~20.116 ± 6.11 μg/m^3^) with a range of 0.78–38.79 ppb (~1.466–72.925 μg/m^3^) in an urban area of Delhi during winter period [8]. The NO_2_ levels in Haryana, India lies in the range of 10.600 μg/m^3^–83.600 μg/m^3^ in sensitive area and between 17.700–117.100 μg/m^3^ in industrial region [6]. Verma et al. [5] reported the concentration of NO_2_ to be 38.240 μg/m^3^ in Lucknow. The concentration of ambient NO_2_ were found to be 97.645 ± 79.034 μg/m^3^, 95.126 ± 52.355 μg/m^3^ and 126.557 ± 83.245 μg/m^3^ in pre monsoon, post monsoon and winter seasons respectively in Burdwan, India [9]. The concentration of nitrogen dioxide was found in range of 0.02–0.08 ppm (~ 37.600–150.400 μg/m^3^) in Pakistan by Ali and Athar [2].

### Source identification

The data of various criteria pollutants are collected and analyzed for identifying the major sources of pollutants over this urban area.

PCA is performed over the data set of two years (June 2013 –May 2015) on seasonal basis for identifying the sources of NO_2_ in four different seasons. The loading of variables on the component are computed for the physical interpretation of the component. An analysis of the PC loadings on the chosen variables allows the identification of the PCs as pollution sources affecting the data and this constitutes the basis of classification. The factors with eigen value more than 1 are chosen for the study as the normalized variables each carry one unit of variance. The numbers of factors (PCs) are selected such that the cumulative percentage variance explained by all the chosen factors is more than 75%. The total variance explained by various variables is 81.897 (in monsoon), 83.017 (in post monsoon), 84.536 (in winter) and 77.389 (in pre monsoon) and are given in Table 1, Table 2, Table 3 and Table 4 respectively. It is observed that vehicular emission is the major source of ambient NO_2_ over the urban region in all the seasons. Therefore, vehicular emission can be used for estimating the future emission scenario over the study area.

**Table 1:** Varimax rotated PC matrix for the criteria pollutants during monsoon season over the urban area.

Monsoon season				
	Industrial emission	Vehicular emission and burning of fossil fuels	Metal processing, metal smelting etc	Miscellaneous sources
PM_10_	0.681	0.212	0.552	0.351
PM_2.5_	0.828	0.110	0.252	0.377
Pb	0.937	-0.212	0.064	-0.076
Ni	0.298	0.286	0.442	0.571
As	0.404	0.304	0.728	0.155
Benzene	-0.024	0.254	-0.090	0.805
Benzo(α)pyrene	0.055	-0.074	0.911	-0.125
CO	0.775	-0.064	0.017	-0.504
NH_3_	0.001	0.856	0.025	0.212
SO_2_	0.275	0.539	0.488	0.210
NO_2_	-0.067	**0.826**	0.364	0.185
O_3_	-0.081	0.908	-0.085	0.044
Eigen value	4.743	2.923	1.146	1.017
Variability (%)	24.734	24.003	19.289	13.872
Cumulative (%)	24.734	48.736	68.025	81.897

**Table 2:** Varimax rotated PC matrix for the criteria pollutants during post monsoon season over the urban area.

Post monsoon			
	Industrial emission	Metal processing, metal smelting etc	Vehicular and biomass burning emission
PM_10 _	0.753	0.388	0.457
PM_2.5_	0.636	0.405	0.530
Pb	0.206	0.925	0.042
Ni	0.482	0.654	0.290
As	0.634	0.356	0.444
Benzene	0.916	0.029	0.266
Benzo(α)pyrene	0.910	0.131	0.225
CO	-0.003	0.862	0.197
NH_3_	0.360	0.020	0.839
SO_2_	0.182	0.365	0.827
NO_2_	0.353	0.097	**0.888**
O_3_	0.537	0.118	0.565
Eigen value	7.301	1.640	1.021
Variability (%)	32.414	22.025	28.578
Cumulative (%)	32.414	54.439	83.017

**Table 3:** Varimax rotated PC matrix for the criteria pollutants during winter season over the urban area.

Winter			
	Industrial emission	Vehicular and biomass burning emission	Miscellaneous sources
PM_10_	0.834	0.097	0.493
PM_2.5_	0.718	-0.312	0.576
Pb	0.809	0.366	0.103
Ni	0.828	0.270	0.143
As	0.552	0.730	0.165
Benzene	0.893	0.205	0.140
Benzo(α)pyrene	0.832	0.432	0.068
CO	0.043	0.515	0.686
NH_3_	0.057	0.866	0.167
SO_2_	0.300	0.070	0.886
NO_2_	0.239	**0.691**	0.463
O_3_	0.281	0.910	-0.106
Eigen value	6.709	2.136	1.300
Variability (%)	38.148	28.327	18.061
Cumulative (%)	38.148	66.475	84.536

**Table 4:** Varimax rotated PC matrix for the criteria pollutants during pre monsoon season over the urban area.

Pre monsoon		
	Industrial emission	Vehicular and biomass burning emission
PM_10_	0.890	0.387
PM_2.5_	0.904	0.109
Pb	0.783	0.270
Ni	0.818	0.089
As	0.607	0.645
Benzene	0.820	0.219
Benzo(α)pyrene	0.660	0.582
CO	0.382	0.628
NH_3_	0.182	0.879
SO_2_	0.811	0.291
NO_2_	0.436	**0.829**
O_3_	0.035	0.951
Eigen value	7.459	1.828
Variability (%)	44.986	32.413
Cumulative (%)	44.986	77.389

### Role of meteorology

**Fig 3:**
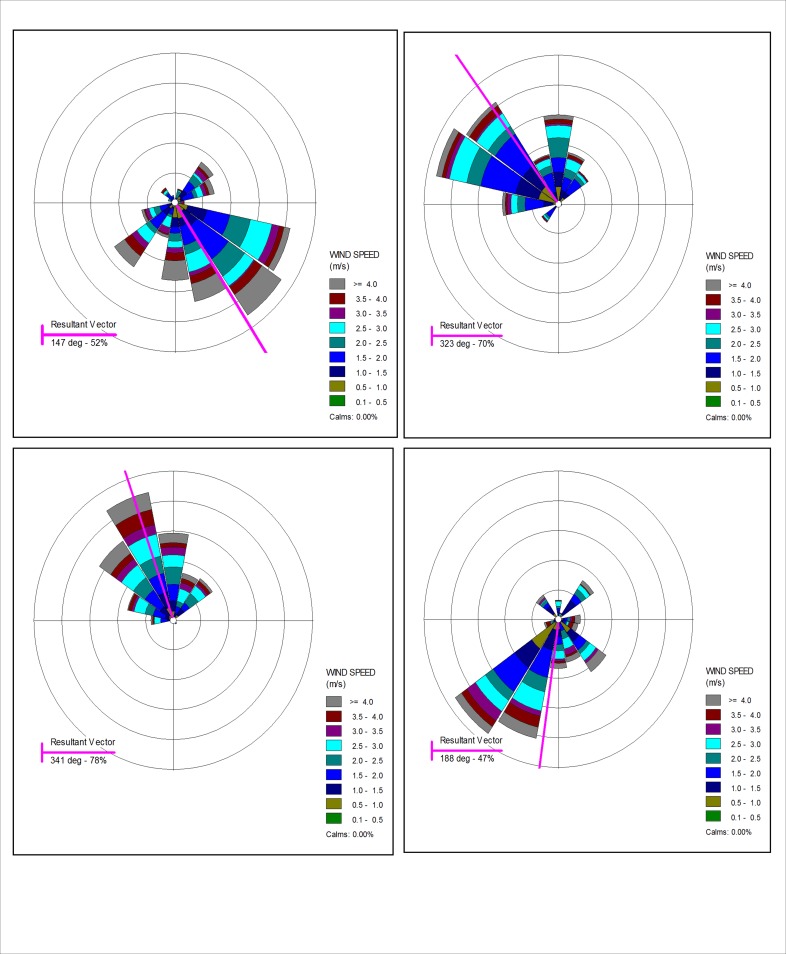
Wind rose diagrams showing the variation of wind speed and wind direction over the urban area in (a) monsoon, (b) post monsoon, (c) winter and (d) pre monsoon seasons.

Local meteorology plays a crucial role in the determination of concentration of NO_2_ over the urban region. High temperature and wind speed results in dispersion and dilution of the air pollutants. It is evident from Fig 3 that wind predominantly flows from the north–west direction in post monsoon and winter seasons. This North-west wind might be responsible for higher concentration of NO_2_ over this urban area during the winter and post monsoon seasons.

#### Correlation analysis

The relationships among the NO_2_ concentration and the meteorological parameters (*i*.*e*. temperature, relative humidity and wind speed) have been explored by correlation analysis (Table 5). It is observed that NO_2_ concentration and temperature hold positive correlation in monsoon (r = 0.803 and p < 0.0001) and pre monsoon (r = 0.194 and p = 0.365) seasons whereas in opposite situation prevails in post monsoon (r = -0.176 and p = 0.411) and winter (r = -0.664 and p = 0.000). Relative humidity bears significant negative relationship with NO_2_ level in all the seasons except monsoon. Wind speed is inversely proportional to NO_2_ level in monsoon (r = -0.238 and p = 0.263), post monsoon (r = -0.606 and p = 0.002), winter (r = -0.532 and p = 0.007) and pre monsoon (r = -0.675 and p = 0.000) seasons. High wind speed helps in dispersion of pollutants thereby diluting the pollutant load near the earth’s surface.

**Table 5:** Correlation coefficients (r) of NO_2_ concentration with respect to meteorological parameters in different seasons over the urban area.

Seasons	Temperature	Relative humidity	Wind speed
Monsoon	0.803	0.279	-0.238
Post monsoon	-0.176	-0.642	-0.606
Winter	-0.664	-0.642	-0.532
Pre monsoon	0.194	-0.436	-0.675

#### Regression analysis

Linear regression analysis is implemented for understanding the influence of individual meteorological parameter on the concentration of NO_2_ by expressing the concentration of NO_2_ as function of temperature, relative humidity and wind speed separately (Table 6). This table suggests that the concentration of NO_2_ is mostly influenced by relative humidity (R^2^ = 0.384) followed by temperature and wind speed.

**Table 6:** Outcomes of regression analysis and equations of NO_2_ and meteorological factors (T, RH and WS).

Meteorological factors	Equations	R^2^
Temperature (T)	173.597–3.858 T	0.204
Relative humidity (RH)	180.804–3.028 RH	0.384
Wind speed (WS)	122.412–19.769 WS	0.061

The regression analysis of NO_2_ *w*.*r*.*t* temperature, relative humidity and wind speed (Table 6) suggest that all the chosen meteorological parameters hold inverse relationship with the concentration of ambient NO_2_. Higher temperature elevates the planetary boundary layer height which in turn dilutes the pollutant level near the earth’s surface.

Multiple regression analysis (step—wise) has been performed on the NO_2_ data set. In the analysis, concentration of NO_2_ (**C**_**NO2**_**)** is assumed as the dependent variable whereas temperature, relative humidity and wind speed are considered as the independent variables. The proposed equation suggests that the chosen meteorological parameters (temperature, relative humidity and wind speed) are responsible for 40.5% variation of the NO_2_ concentration. The constructed equation is as follows

$$C_{NO_{2}}=208.711-0.633*T-2.646*RH-11.32*WS\;\left(R^{2}=0.405\right)$$ (5)

### Futuristic emission scenario

Vehicular emission is found to be the major source of NO_2_ in the chosen urban atmosphere. So the NO_x_ emitted from the vehicular exhaust is used for portraying the futuristic emission scenario. NO_X_ generally refers to the mixture of NO and NO_2_ and NO rapidly get converted into NO_2_ in the atmosphere. On this basis, the model generated data of NO_X_ have been compared with the primary and secondary data of NO_2_ in this work.

The NO_x_ emission due to vehicles in the chosen urban area is estimated using registered vehicular data of RTO (Regional Transport Office), pollutant emission factors recommended by ARAI (Automotive Research Association of India), Pune and roads length for the present scenario taking 2014 as base year. On the basis of the report, the average annual growth rate of vehicles in this area is found to be 12.05%. Considering 2014 as the base year, the number of different types of vehicles has been calculated for the year 2024 and 2034. Table 7 shows the number of different vehicles in 2014, 2024 and 2034 as well as the emission factors of NO_X_ according to ARAI [25] for different types vehicles.

**Table 7:** Various types of vehicles with emission factors of NO_X_ according to ARAI (2007) and the number of vehicles in 2014, 2024 and 2034 over the urban area.

				
	2014	2024	2034	Emission Factor (gkm^-1^)
Vehicle Type	No. of Vehicles	No. of Vehicles	No. of Vehicles	
**Two Wheelers (Petrol)**	218293	662719	2035682	0.15
**Personal Cars (Petrol)**	21137	64171	197115	0.09
**Personal Cars (Diesel)**	2047	6214	19088	0.28
**Three Wheelers (Petrol)**	8842	26843	82453	0.16
**Buses (Diesel)**	1493	4533	13925	6.53
**Heavy Commercial Vehicles (Diesel)**	531	1613	4955	9.3
**Light Commercial Vehicles (Diesel)**	7240	21980	67516	2.12

Using obtained emission scenario (Table 7) and WRF generated surface and upper air data, the ground level concentrations of NO_X_ over the study area in four different seasons (winter, pre monsoon, monsoon and post monsoon) are computed by AERMOD (Table 8). It is observed that the concentrations of NO_X_ in 2014 obtained as per ARAI norms have exceeded the 24 h NAAQS permissible limit (*i*.*e*.80 μg/m^3^) in winter (116.005 μg/m^3^), pre monsoon (115.50 μg/m^3^) and post monsoon (110.96 μg/m^3^) seasons. The predicted values for 2024 and 2034 are quite high. It might be due to the fact average annual growth rate of the vehicle has been considered but the number of vehicles de-registered every year has not been taken into consideration due to unavailability of data. Moreover, use of modern technology and introduction of better control measures in automobiles are expected to improve efficiency of engines and reduce the emission rate of NO_X_ thereby decreasing the concentration of ambient NO_X_ in the coming year.

**Table 8:** NO_X_ concentration 2014, 2024 and 2034 by using different emission scenarios over the urban area.

Scenario	Year	According to	Concentration of NO_x_(μg/m^3^)in different seasons			
			Winter	Pre monsoon	Monsoon	Post monsoon
Present	2014	ARAI Norms	116.005	116.50	71.68	110.96
Future	2024	ARAI Norms	329.55	331.68	200.29	321.23
	2034	ARAI Norms	907.08	907.78	550.24	883.76

### Comparison of model output with field observation

**Fig 4:**
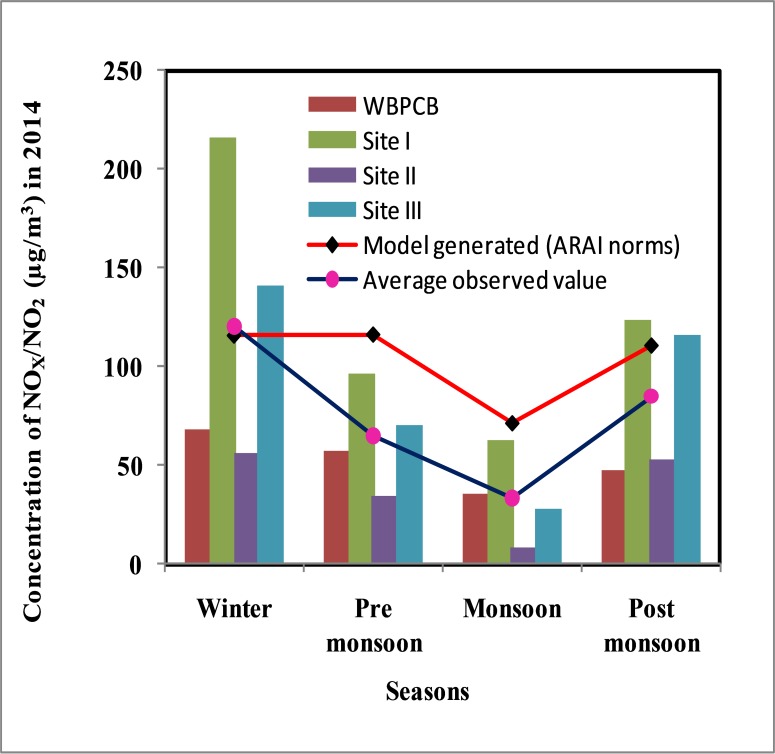
Comparative study of the concentration of NO_X_/NO_2_ (μg/m^3^) in 2014 over the urban area.

The model generated concentration of NO_X_ (μg/m^3^) for the year 2014 is compared with the archived data of NO_2_ concentration obtained from WBPCB (Durgapur unit) and the primary data recorded at three different sites during the field work in different seasons of 2014 (Fig 4).

The comparative study reflects the concentrations of NO_X_ as per ARAI norms are higher than that of data recorded by WBPCB in all the seasons of 2014 but fair agreement exists between model generated NO_X_ data (as per ARAI norms) and primary data obtained from three different sites (especially the site with high vehicular density, Site I). It is found that the model generally overestimates the concentration of NO_2_ over the urban area in all the seasons (except winter season).

## Conclusion

The present study highlights the spatial distribution of NO_2_ in different seasons over the urban area. The spatial and temporal variation of NO_2_ level over the urban area is a manifestation of combined effects of emission sources, meteorology and planetary boundary layer characteristics. Prediction of NO_X_ concentration using coupled WRF–AERMOD model shows encouraging results. This works imparts an idea of future emission scenario of NO_2_ over the urban area. Although source identification of NO_2_ over the urban area shows that vehicular emission is the major source of NO_2_, but the industrial emission of this region also influences the ambient concentration of NO_2_. It is observed that model estimated concentrations of NO_2_ exceed the observed average concentrations in all the seasons (except winter). An understanding the season–wise sources and sinks of NO_2_ over this study area might improve the obtained result. Therefore, preparation of comprehensive emission inventory by considering all available polluting sources (especially including the industrial emission) of this urban area as well as incorporation of data of yearly de-registered vehicles will portray a more realistic scenario.
